# Breakpoints in ventilation, cerebral and muscle oxygenation, and muscle activity during an incremental cycling exercise

**DOI:** 10.3389/fphys.2014.00142

**Published:** 2014-04-11

**Authors:** Sebastien Racinais, Martin Buchheit, Olivier Girard

**Affiliations:** ^1^Research and Education Centre, Aspetar, Qatar Orthopaedic and Sports Medicine HospitalDoha, Qatar; ^2^Sport Science Department, Myorobie AssociationMontvalezan, France; ^3^Department of Physiology, Faculty of Biology and Medicine, Institute of Sport Sciences, University of LausanneLausanne, Switzerland

**Keywords:** maximal oxygen consumption, ventilatory threshold, electromyography, near infra-red spectroscopy, cycling

## Abstract

The aim of this study was to locate the breakpoints of cerebral and muscle oxygenation and muscle electrical activity during a ramp exercise in reference to the first and second ventilatory thresholds. Twenty-five cyclists completed a maximal ramp test on an electromagnetically braked cycle-ergometer with a rate of increment of 25 W/min. Expired gazes (breath-by-breath), prefrontal cortex and *vastus lateralis* (VL) oxygenation [Near-infrared spectroscopy (NIRS)] together with electromyographic (EMG) Root Mean Square (RMS) activity for the VL, *rectus femoris* (RF), and *biceps femoris* (BF) muscles were continuously assessed. There was a non-linear increase in both cerebral deoxyhemoglobin (at 56 ± 13% of the exercise) and oxyhemoglobin (56 ± 8% of exercise) concomitantly to the first ventilatory threshold (57 ± 6% of exercise, *p* > 0.86, Cohen's *d* < 0.1). Cerebral deoxyhemoglobin further increased (87 ± 10% of exercise) while oxyhemoglobin reached a plateau/decreased (86 ± 8% of exercise) after the second ventilatory threshold (81 ± 6% of exercise, *p* < 0.05, *d* > 0.8). We identified one threshold only for muscle parameters with a non-linear decrease in muscle oxyhemoglobin (78 ± 9% of exercise), attenuation in muscle deoxyhemoglobin (80 ± 8% of exercise), and increase in EMG activity of VL (89 ± 5% of exercise), RF (82 ± 14% of exercise), and BF (85 ± 9% of exercise). The thresholds in BF and VL EMG activity occurred after the second ventilatory threshold (*p* < 0.05, *d* > 0.6). Our results suggest that the metabolic and ventilatory events characterizing this latter cardiopulmonary threshold may affect both cerebral and muscle oxygenation levels, and in turn, muscle recruitment responses.

## Introduction

Cardiopulmonary adjustments to exercise have been the main focus of exercise physiologists for decades. Lavoisier mentioned the necessity of respiration to allow combustion as early as 1777 (Lavoisier, 1777) and did the first attempt to measure pulmonary gas exchange at rest and during exercise (Mitchell and Saltin, 2003). One and half century later, Hill and Lupton defined and precisely measured the maximum oxygen uptake ($\dot{V}$*O*_2max_) in exercising human (Hill and Lupton, 1923); and, another half-century latter, several studies determined the first and second ventilatory thresholds (V-T1 and V-T2, respectively) in athletes based on non-linear respiratory responses to an increase in exercise intensity (Davis et al., 1979; Kinderman et al., 1979). However, the exact nature of the underpinning factors that determine the occurrence of the ventilatory thresholds are still debated, and likely include an increased blood concentration of muscle metabolites directly acting on the respiratory centers, and/or the activation of nervous sensory pathways originating in contracting muscles (Dempsey et al., 1995). Importantly, the cascade of physiological events in the organs responsible for locomotion (e.g., leg muscles and brain) associated with these non-linear respiratory changes remains unclear.

In 2003, Hug et al. reported that the ventilatory thresholds during an incremental cycling test were generally accompanied by one (and sometimes two) electromyographic thresholds (EMG-T) as manifested by a non-linear increase in the activity of the *vastus lateralis* (VL). The existence of EMG-T was later confirmed in the VL (Osawa et al., 2011) and other quadriceps muscles as *rectus femoris* (RF) and *biceps femoris* (BF) (Rupp and Perrey, 2008) and may be attributable to an increased recruitment of fast twitch motor units to maintain the required energy supply for contraction (Moritani and deVries, 1978). In the meantime, the near-infrared spectroscopy (NIRS) technique was applied to non-invasively determine the changes in tissue oxygenation in athletes performing incremental exercise (Bhambhani et al., 1997, 1998; Perrey, 2008). Several studies reported that muscle deoxygenation follows a sigmoidal profile with a slowdown or plateau marking a NIRS threshold toward the end of an incremental exercise (Ferreira et al., 2007; Legrand et al., 2007; Boone et al., 2010). These patterns in oxy/deoxyhemoglobin have been attributed to changes in capillary and venous oxygenation and tissue myoglobin O_2_ saturation, which fluctuate in relation to PO_2_, blood acidosis and exercise-related balance between O_2_ delivery and utilization at the site of measurement (Belardinelli et al., 1995; Bhambhani et al., 1997). However, the timing of the different muscle oxygenation and electrical activity responses relatively to each other remains conflicting. Whereas Rupp and Perrey (2008) reported that EMG-T occurred before V-T2 and therefore before the stabilization in muscle total hemoglobin, Osawa et al. (2011) reported that EMG-T occurred after the attenuation (i.e., a slowdown or plateau point) in muscle deoxygenation (Osawa et al., 2011). The subjectivity of threshold determination in some studies (e.g., Rupp and Perrey, 2008) or the fact that some other studies report discrete data during the incremental exercise (e.g., Subudhi et al., 2007) make difficult to conclude on the sequence of events occurring at muscle level. A simultaneous and objective determination of the non-linear changes in both muscle EMG and oxygenation during an incremental exercise is therefore required.

In addition, applying cerebral NIRS during an incremental exercise has recently allowed to show an increase followed by a decrease in cerebral oxyhemoglobin (Subudhi et al., 2007; Rooks et al., 2010) when reaching VT-2 (Bhambhani et al., 2007; Rupp and Perrey, 2008; Oussaidene et al., 2013). In fact, while Bhambhani et al. (2007) found a significant 30-s delay in cerebral oxygenation threshold in comparison with respiratory compensation threshold, this difference was non substantial practically, i.e., standardized difference or Cohen's *d* < 0.1. These changes in cerebral oxygenation might be linked to these in PaCO_2_, which plays an important role in regulating cerebral blood flow (Madden, 1993). In fact, an increase in PaCO_2_ induces cerebral vasodilation thereby increasing blood flow, while a decrease in PaCO_2_ has the opposite effect. While PaCO_2_ generally increases after V-T1, it decreases abruptly after V-T2 (Wasserman, 1986); so the cerebral oxygenation does (Bhambhani et al., 2007). In addition, Rupp and Perrey (2008) recorded for the first time the cerebral NIRS responses and the muscle EMG and NIRS responses concomitantly during an incremental exercise. However, in their study the authors do not report direct comparison of objectively calculated EMG non-linear changes relatively to the NIRS non-linear changes. Therefore, how breakpoints in cerebral and muscle oxygenation, and muscle activity (i.e., EMG) are linked during a ramp task remain to be determined.

Consequently, in order to better understand the possible associations between the different physiological variables' thresholds occurring within the different organs, the aim of this study was to locate the breakpoints in cerebral and muscle oxygenation, and muscle electrical activity during a ramp exercise in relation to the first and second ventilatory thresholds. Based on previous findings supporting a systemic and generalized cascade of physiological events in the different organs (i.e., muscle and brain) (Dempsey et al., 1995; Bhambhani et al., 1997; Hug et al., 2003; Rooks et al., 2010), we expected cerebral and muscle oxygenation, and muscle activity thresholds to occur concomitantly to the ventilatory thresholds.

## Methods

### Participants

25 cyclists completed the study after providing a written informed consent. Their characteristics were as follow (mean ± SD): age 37 ± 8 years, weight 78 ± 13 kg, height 178 ± 8 cm, training 7 ± 3 h per week. None of the participants suffered from injuries at the time of the experiment and they were asked to avoid all vigorous activity for the 24 h preceding the trial. The procedures complied with the Declaration of Helsinki regarding human experimentation and was approved by the institutional review board.

### General procedure

Each participant performed an incremental cycling test on an electromagnetically braked cycle ergometer (Excalibur, Lode, Groningen, The Netherlands), which was adjusted to each participant's specifications and with the feet securely strapped on the pedal. Workload increased at a ramped rate of 25 W.min^−1^ until subjects reached exhaustion, as indicated by volitional cessation of exercise, or failure to maintain a pedal cadence of 70 rpm despite strong verbal encouragement. Testing was performed under standard environmental conditions (temperature ~22°C and relative humidity ~40%). Systemic [Heart rate (HR) and expired gazes], electromyographic (EMG activity of the lower limb), and muscle/cerebral oxygenation responses were continuously recorded.

### Cardiopulmonary responses

Expired gazes were collected by a mask enclosing both the mouth and nose and recorded by a breath by breath analyzer (Cosmed Quark b^2^, Rome, Italy) for calculation of ventilation ($\dot{V}$E), O_2_ consumption ($\dot{V}$*O*_2_) and CO_2_ production ($\dot{V}$*CO*_2_). HR was continuously recorded using a chest strap (Polar, Electro OY, Kempele, Finland). The maximal oxygen consumption ($\dot{V}$*O*_2max_) was defined according the following criteria: (1) a leveling off in $\dot{V}$*O*_2_, defined as an increase of less than 1,5 ml/kg/min^−1^ despite progressive increases in exercise intensity, (2) a respiratory exchange ratio higher than 1.10, (3) a final HR above 90% of the age related maximum, (4) apparent exhaustion at the termination of the test, and (5) the incapacity to maintain a pedal cadence of 80 rpm. The first ventilatory threshold (V-T1) was determined using the criteria of an increase in $\dot{V}$E/$\dot{V}$*O*_2_ with no increase in VE/$\dot{V}$*CO*_2_ and departure from the linearity of $\dot{V}$E, whereas V-T2 corresponded to an increase in both $\dot{V}$E/$\dot{V}$*O*_2_ and $\dot{V}$E/$\dot{V}$*CO*$\dot{V}$*CO*_2_ (Wasserman and McIlroy, 1964). All assessments of the V-T1 and V-T2 were made by visual inspection of graphs of time plotted against each relevant respiratory variable (averaged on 30 s). All assessments were done independently by two experienced exercise physiologists. In case of divergent results, they revaluated the original assessments together to agree on a consensus.

### Prefrontal cortex and muscle oxygenation responses

Uninterrupted measurements of cerebral and muscle tissue oxygenation trends were obtained via NIRS (Oxymon MkIII, Artinis, The Netherlands). One NIRS emitter-detector pair was placed over the left prefrontal lobe, between Fp1 and F3 (international EEG 10–20 system). A second emitter-detector pair was placed on the distal part of the right VL (approximately 15 cm above the proximal border of the patella). Spacing between optodes was fixed at 45 mm using a black, plastic spacer held in place via double-sided tape. Skinfold thickness at the site of application of the NIRS probe was determined before the testing session and was less than half the distance between the source of the detector (i.e., 45 mm). The electrode spacers were also secured with black elastic bandage to reduce the intrusion of extraneous light. A modified form of the Beer–Lambert Law was used to calculate micromolar changes in tissue oxyhemoglobin [O_2_Hb] and deoxyhemoglobin [HHb] across time using received optical densities from two continuous wavelengths of NIRS light (763 and 855 nm). Differential path length factors were fixed at 5.93 for cerebral (van der Zee et al., 1992) and at 3.83 for muscle (Kowalchuk et al., 2002) tissues. NIRS data were acquired at 10 Hz.

### Electromyographic responses

Surface electromyographic signals (EMG) were recorded from two quadriceps muscles (VL and RF) and one hamstring muscle (BF) using MP35 hardware (common-mode rejection ratio, CMRR: 85 dB) and dedicated software (BSL Pro Version 3.6.7, Biopac Systems Inc., Santa Barbara, CA). Bipolar Ag/AgCl electrodes (Ambu Blue sensor T, Ambu A/S, Denmark) with a diameter of 9 mm and an interelectrode distance of 3 cm were placed on the muscle belly after preparation of the skin to minimize impedance (i.e., shaving, light abrasion and alcohol cleaning to remove surface layers of hair, dead skin, and oil). A ground electrode was placed on the right wrist. The myoelectric signal was amplified (gain = 1000×, bandwidth = 470 Hz), filtered (30–500 Hz), and the frequency of data collection was 2000 Hz.

### NIRS and EMG data analyses

All NIRS and EMG signals were normalized to 100 points (1 point per percent of exercise duration). To facilitate the comparison of the kinetics of variation between muscles, the root mean square (RMS) of the raw signal was calculated for each 1% of the exercise and was normalized by the maximal activity of the same muscle recorded during the incremental test for each participant. Data were analyzed via an automatized-computerized routine developed under LabVIEW (National Instruments, TX). This routine was based on the method previously described by Osawa et al. (2011) to objectively determine the presence of non-linear changes in the RMS (VL, RF, and BF) and NIRS (HHb and O_2_Hb, both muscle and cerebral) signals.

For all NIRS and EMG signal, a linear regression was applied from the beginning of the signal to a point “*n*.” A second linear regression was applied from the point “*n* + 1” to *n* = 80 (to avoid any effects of a potential second threshold on the first determination). The error sum of squares of each regression line was calculated. These steps were repeated for all “*n*” from *n* = 2 to *n* = 78. The “*n*” presenting the smallest sum of the error sum of squares of the two lines was determined as the first threshold. Thereafter the second threshold was determined following the same procedure with “*n*” ranging from the first threshold to *n* = 98 (i.e., 2 points before the end of the signal). If the analyzed signal did not present any thresholds more marked than the initial response to exercise (i.e., the routine showed that the best adjustment was obtain with a breakdown point below 15% of the exercise), the threshold determination was considered as unsuccessful.

### Statistical analyses

Normality of the data was tested using Shapiro-Wilk test and non-normal data were log transformed. Based on the limitation of an inconsistent number of observations (see results), ANOVAs could not be performed and a linear mixed model was performed to determine the effect of each variable on threshold locations (expressed in % of the ramp test duration). This procedure has ability to accommodate missing data (Krueger and Tian, 2004). *Post-hoc* comparisons were performed between each variable with Least Square Difference correction. The level of statistical significance was set at *p* < 0.05. Effect size were calculated as Cohen's *d* and classified as moderate for 0.6 < *d* = 1.2 and large for *d* > 1.2 (Hopkins et al., 2009).

## Results

### Systemic responses

The average $\dot{V}$*O*_2max_ was 53 ± 8 ml.min^−1^.kg^−1^, reached after 13 min 50 s (± 2 min 13 s) at a maximal power output of 346 ± 56 W. The V-T1 and V-T2 were determined at 57 ± 6% (199 ± 45 W) and 81 ± 6% (280 ± 51 W) of test duration, respectively.

### Deoxygenation responses

**Figure 1:**
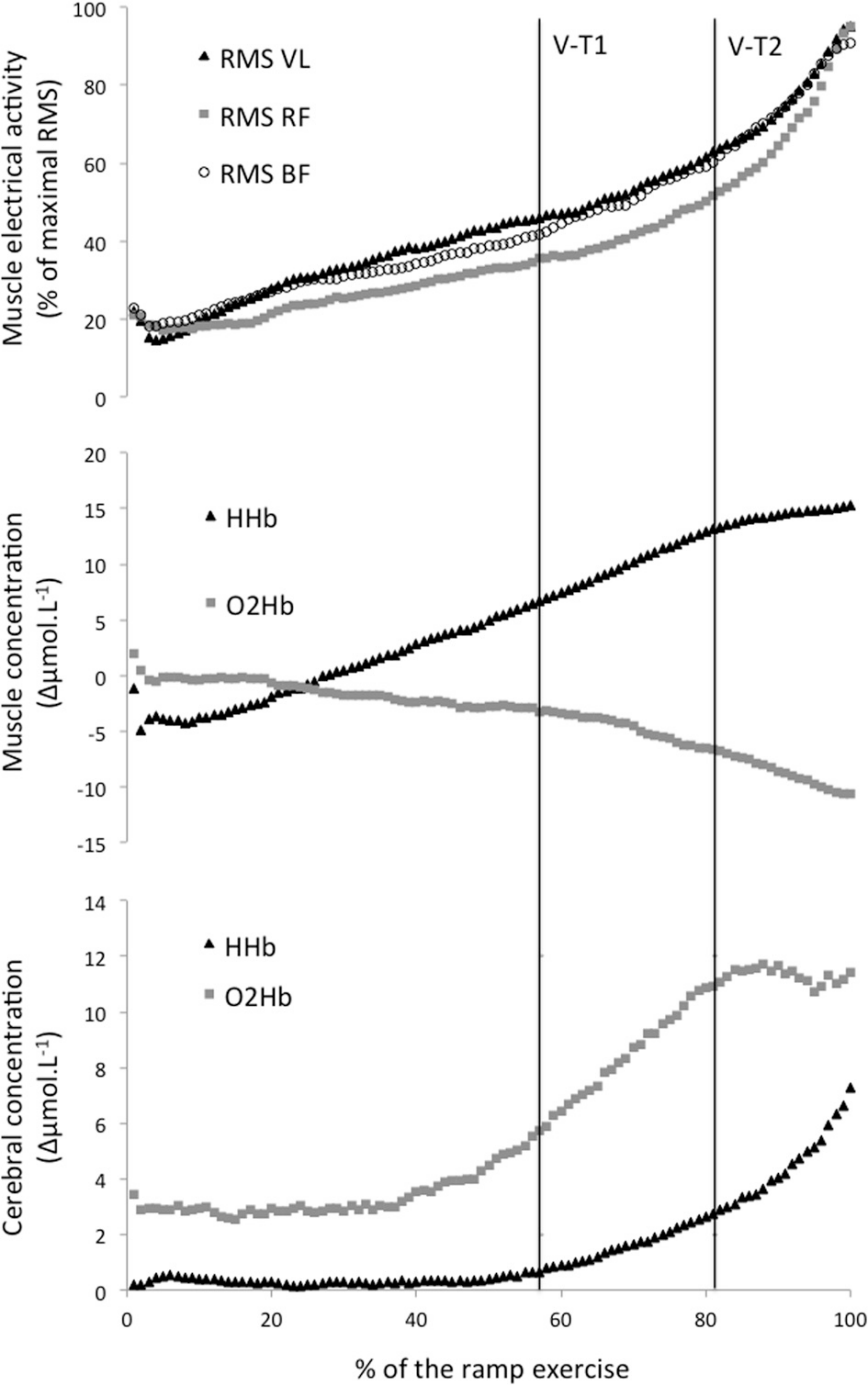
**Average electromyographic (top graph) and Near Infrared Spectroscopy (middle graph: muscle, bottom graph: cerebral) parameters during an incremental ramp exercise**. V-T1 and V-T2, first and second ventilatory threshold. RMS, root mean square; VL, *vastus lateralis*; RF, *rectus femoris*; BF, *biceps femoris*; HHb, deoxyhemoglobin; O_2_Hb. oxyhemoglobin.

**Figure 2:**
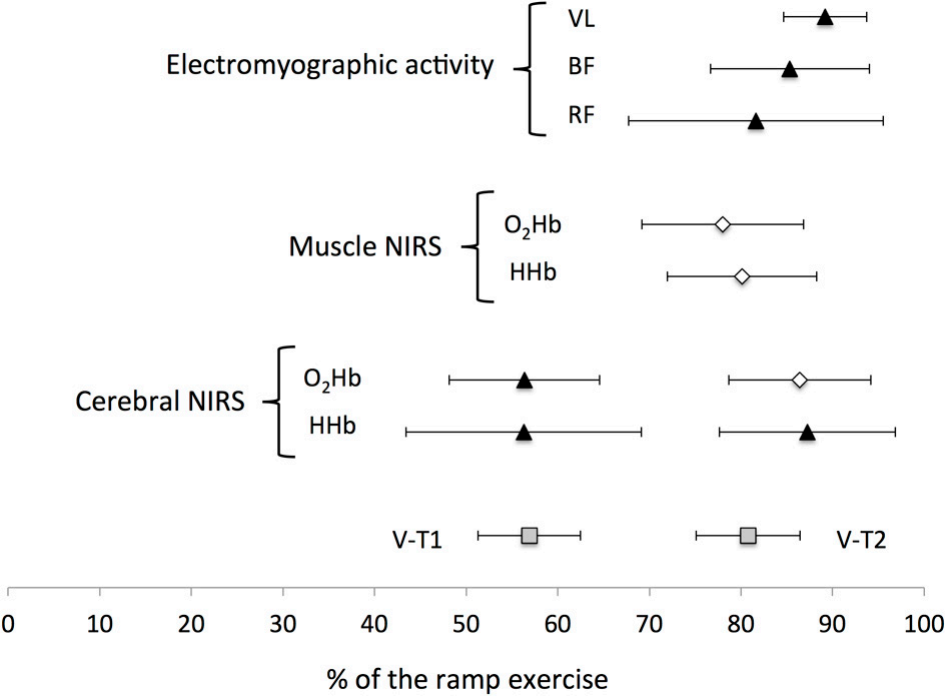
**Mean (±SD) location of the non-linear changes occurring during a ramp exercise**. V-T1 and V-T2, first and second ventilatory thresholds. NIRS, Near-infrared spectroscopy; VL, *vastus lateralis*; BF, *biceps femoris*; RF, *rectus femoris*; HHb, deoxyhemoglobin; O_2_Hb, oxyhemoglobin.

Two cerebral thresholds were determined in 80% of the recordings. Cerebral HHb increased non-linearly during the ramp exercise with a first and second threshold determined at 56 ± 13 and 87 ± 10% of the ramp exercise, respectively. Conversely, the cerebral O_2_Hb follow a sigmoidal variation with a non-linear increase around 56 ± 8% of the ramp exercise but a plateau/decrease around 86 ± 8% of the ramp exercise (Figures 1, 2). The location of the first threshold in cerebral HHb and O_2_Hb was not statistically/substantially different than V-T1 (*p* > 0.86, *d* < 0.1) whereas, the second threshold in cerebral HHb and O_2_Hb occurred moderately (*d* > 0.8) and significantly (*p* < 0.02) latter than V-T2 by 7 ± 12% (*p* = 0.016) and 7 ± 9% (*p* = 0.017), respectively.

Only one threshold could be determined on the muscle oxygenation parameters. In 64% of the recordings, muscle O_2_Hb displayed a non-linear decrease at 78 ± 9% of the ramp exercise and the increase in muscle HHb was attenuated from 80 ± 8% of the ramp exercise in 40% of the recordings (Figures 1, 2). These changes occurred very largely (both *d* > 2.9) and significantly latter that V-T1 (both *p* < 0.001) at a time not statistically different than V-T2 (both *p* > 0.15, *d* < 0.4).

### Electromyographic responses

Only one EMG-T could be consistently determined (in 60 to 88% of the recordings, depending on the muscle). The EMG-T for the VL, RF, and BF occurred at 89 ± 5, 82 ± 14, and 85 ± 9% of the ramp exercise, respectively (Figure 1). The EMG-T for VL occurred largely after V-T2 (+10 ± 7%, *d* = 1.7, *p* = 0.004), after the attenuation (i.e., level off) in muscle HHb increase (+7 ± 9%, *d* = 1.4, *p* = 0.014) and after the non-linear decrease in muscle O_2_Hb (+6 ± 17%, *d* = 1.7, *p* = 0.006).

The normalized RMS activity for RF was significantly lower than for both VL and BF from 14–15 to 95–96% of the ramp exercise (all *p* < 0.05).

## Discussion

In the current study we simultaneously located for the first time the break points of cerebral and muscle oxygenation, and muscle electrical activity during a ramp exercise in reference to V-T1 and V-T2. Our data showed that while the non-linear changes in cerebral oxygenation were concomitant to both V-T1 and V-T2, the changes in muscle oxygenation and electrical activity occurred only concomitantly to or after V-T2 (Figure 2).

### De-oxygenation responses and relationship with other physiological responses

The current data showed non-linear increases in cerebral oxyhemoglobin and deoxyhemoglobin at a time that was not statistically different from V-T1 (Figure 2). These changes are in agreement with previous findings (Rooks et al., 2010), and might be related to a local increase in cerebral blood flow in response to a rise in PaCO_2_. However, there was no clear non-linear variation in any muscle oxygenation variables nor in muscle electrical activity at this intensity. The changes in muscle oxygenation and electrical activity occurred at intensities significantly higher than V-T1, close to V-T2 (Figure 2). For example, in the current study, the EMG-T were identified in the RF, BF, and VL at 82, 85, and 89% of the ramp exercise respectively, a result similar to the 85% reported by Hug et al. (2003). However, whereas Rupp and Perrey (2008) reported that the non-linear increase in RMS EMG occurred before V-T2, our data showed that the EMG-T for BF and VL occurred after V-T2 (*d* > 0.6, Figure 2). This discrepancy might be attributed to methodological differences such as the automatic threshold determination used in the current study or the utilization of a ramp protocol vs. an incremental step protocol. Conversely, these studies suggest that a non-linear increase in EMG of the leg muscle occurs close to V-T2. The attenuation in muscle HHb observed in the current study is also in accordance with the literature reporting a slowdown or plateau in muscle HHb toward the end of an incremental exercise (Ferreira et al., 2007; Legrand et al., 2007; Boone et al., 2010). When comparing the different muscle responses, our data confirmed the observation from Osawa et al. (2011), and showed that the attenuation in muscle HHb increase preceded by 7 ± 9 and 9 ± 12% the non-linear increase in VL and BF RMS activities. In addition, the current data showed that the non-linear increase in VL RMS occurred largely (*d* = 1.7) latter than the non-linear decrease in VL O_2_Hb by 6 ± 17%. Collectively, these observations suggest that oxygenation changes at muscle level might partly play a role in driving the EMG-T. However, the standard deviations of the average differences were large and overlapping zero and the change in RMS activity was muscle dependent. Interestingly, our results suggest that the changes in EMG activity of each muscle are probably dependent of the recruitment strategy of the synergist muscles (see below).

Cerebral oxygenation responses displayed, non-linear changes significantly after V-T2. For example, there was a non-linear increase in cerebral HHb occurring 7 ± 12% latter than V-T2, suggesting that cerebral oxygenation changes during an incremental exercise might be driven by the systemic changes. Present results contrast however with previous findings (Bhambhani et al., 2007; Rupp and Perrey, 2008; Oussaidene et al., 2013), where the cerebral oxygenation threshold occurred concomitantly to VT-2. This non-linear increase in cerebral deoxygenation (increased HHb and decreased O_2_Hb) occurred at a time similar to the non-linear increases in EMG activity (Figure 2). This confirms that the changes in muscle recruitment strategies might be related to changes in cerebral oxygenation (Bhambhani et al., 2007). In fact, the reduction in PaCO_2_ generally observed after VT-2 induces cerebral vasoconstriction and reduces blood flow. This may result in reduced neuronal activation, and in turn, neural drive and muscle activation (Noakes, 2000). Given that the current results showed that the decrease in cerebral oxygenation and the non-linear increase in RMS VL and RMS BF were all located significantly later than V-T2, this suggests that the EMG activity response might be triggered by systemic changes.

### Electromyographic responses in different leg muscles

As displayed in Figure 1, the kinetic of increase in RF was slightly different from both VL and BF muscles. As a consequence, activation of the RF (relative to its maximum activity during the test) became lower than the activity recorded for both BF and VL muscles after the early stages of the incremental test. This lower RF EMG activity was observed up to 95% of total test duration due to a large increase in RF EMG activity occurring near exhaustion. The discrepancy between RF and BF behaviors could be related to the different muscle group considered (antagonist of each other). Previous studies have reported that the EMG activity of the BF decreases with fatigue during repeated cycling sprints (Hautier et al., 2000; Racinais et al., 2007). This decrement has been interpreted as an improvement in intramuscular coordination to partly compensate for muscle fatigue (Hautier et al., 2000). The current data add to these observations that the EMG activity of the BF increases in the last stages of the incremental exercise (Figure 1), probably when the BF is acting as an agonist muscle. Indeed, the BF could be considered as an agonist muscle during the upstroke of the pedal revolution if the cycloergometer is equipped with toe-clips, as in the present study (Racinais et al., 2007).

The discrepancy between two quadriceps muscles (i.e., VL and RF) could be related to the different muscle function/action. Indeed, VL is a mono-articular muscle participating to the leg extension whereas RF is a bi-articular muscle participating to both leg extension and hip flexion. Therefore, a higher RF activation has previously been considered as a sign of both pulling and pushing pedals and a weaker activation a sign in a decrease in one or both of this action (Hug et al., 2004). The results of the current study as displayed in Figure 1 suggest that, at moderate intensity, the VL and BF provide most of the effort for respectively pushing and pulling; whereas, when intensity increases above V-T2, there is a large increase in RF activity.

### Limitations

The first limitation on the present study is that ventilatory responses could not be analyzed through the same automatized method than NIRS and EMG signals due to the inherent lower sampling frequency (breath-by-breath). However, they have been analyzed through “conventional” methods (Wasserman and McIlroy, 1964; Wasserman, 1986) and are therefore comparable to the literature findings. Secondly, the NIRS and EMG analyses were limited to a restricted number of sites (left prefrontal lobe and VL for NIRS, and VL, RF, and BF for EMG); whether their respective responses are representative enough of/linked with the systemic, cardiorespiratory responses is unknown. Future studies might consider multi-sites recording electrodes (e.g., multichannel NIRS and array of EMG electrodes). Given the important role played by the glutei or plantar flexors in cycling (Hug and Dorel, 2009), their activity could also be recorded in future studies as their behavior could differ or be differently related to the present NIRS measurements. The inability to detect an EMG threshold could originate (among other factors) from putative compensations between synergistic muscles, or an inhomogeneous distribution of EMG activity that could exist within a muscle. Similar limitations apply to the NIRS measures, where the examination of other locations within the same tissues (large within tissue heterogeneity) (Koga et al., 2007) or different muscles could have led to different results. Additionally, while the lack of PaCO_2_ data (or PETCO_2_, that could be used a as proxy (Bhambhani et al., 2007) might limit the interpretation of the possible mechanisms responsible for the changes in cerebral oxygenation, this was unlikely to affect the main conclusions of the present study, i.e., the unmatched occurrence of some of the variable thresholds.

## Conclusions

The aim of this study was to locate the breakpoints of cerebral and muscle oxygenation, and muscle electrical activity during a ramp exercise in reference to the first and second ventilatory thresholds. Our data showed that cerebral oxygenation parameters displayed non-linear changes at both V-T1 and V-T2. Conversely, VL muscle oxygenation and electrical activity displayed thresholds at or after V-T2 only. The changes in cerebral oxygenation and in BF, VL electrical activity occurred moderately (*d* > 0.6) and significantly (*p* < 0.05) latter than V-T2, suggesting that the metabolic and ventilatory events characterizing this latter cardiorespiratory threshold may affect both cerebral and muscle oxygenation levels, and in turn, muscle recruitment responses.

## Author contributions

Sebastien Racinais, Martin Buchheit, and Olivier Girard have contributed to the conception of the work, the data acquisition and interpretation. They revised and approved the manuscript. In addition, they agreed to be accountable for all aspects of the work in ensuring that questions related to the accuracy or integrity of any part of the work are appropriately investigated and resolved.

### Conflict of interest statement

The authors declare that the research was conducted in the absence of any commercial or financial relationships that could be construed as a potential conflict of interest.
